# Observations on Select Subjects in Surgery

**Published:** 1805-01-01

**Authors:** 


### Observations on select Subjects in Surgery,
### By Mr. Simmons.

( Continued from Vol. x. pp. 1—10. )

In your Number for March (p. 210) are contained some judicious remarks by your very respectable Correspondent, Mr Hey, of Leeds, relative to the disease which he has denominated 'Fungus Hæmatodes,' in reply to my observations on a case inserted in your Number for July preceding. That case I had assumed as a more simple specimen of the 'Fungus Hæmatodes;' and from it had hoped to deduce, as far as a single case would go, a satisfactory pathology of the more intricate forms of that disease. Mr. Hey, however, is of a different opinion; so that rather than again enter, perhaps prematurely, on the subject, I shall wait the result of more ample experience. Meanwhile I may be permitted to observe, that, if my opinions are erroneous, instead of one, chirurgical nosology will be enriched by the accession of three new diseases; namely, the 'uncommon disease,' of Mr. Russell; the 'spongoid inflammation,' of Mr. Burns; and the 'Fungus Hæmatodes,' of Mr. Hey. The latter gentleman (p. 213) is indeed of opinion, that the 'spongoid inflammation,' and fungus hæmatodes,' are *one* and the same disease, although it may probably be thought by some, that his own brief mention of those two diseases, in the paper I am quoting, has disproved their identity. The contiguous lymphatic glands, and through these the system at

 large, large, become contaminated, in the 'spongoid inflammation,' a consequence unknown in the 'fungus hæmatodes;' yet we all know that an ulcerated surface is most favourable to absorption. Were this distinction unimportant in practice, I should be less disposed to insist upon it here; but its influence over the curative means is very decisive. In the 'fungus hæmatodes' complete success may be expected, at any period of the disease, by the entire removal of the local affection; but in the 'spongoid inflammation,' after signs of absorption are manifested, any operation is unavailing. In this peculiarity it is analogous to cancer and other morbid poisons.

I join with the profession in general, in expressing my obligations to Mr. Hey for the very valuable present of his publication, and particularly for his renewed attention to the subject before me. If at any time I should dissent in opinion from so respectable an authority, it will always be done in distrust of my own judgment, and with an earnest desire to profit by his judicious corrections.

### On Tinea Capitis.

This is a loathsome and obstinate disease. It is confined to no sex nor age, though most common at an early period; yet, in some rare instances, it is of permanent duration, and continues through life. To acquire a remedy for an inveterate disease, is at all times desirable; and to be able to subdue one of a nature so intractable, is an object greatly to be wished; yet the several means that have been recommended in tinea capitis, are not only both harsh and offensive, as an enumeration of them (which would be superfluous here) would amply testify; but they are far from being speedily or durably effectual.

It is enough, for my present purpose, to propose a method of treating it, not only free from these several objections, but in itself simple, mild, and unattended with pain in its application, and at the same time generally efficacious. I should have felt a delicacy in thus announcing any intimation of my own; but the facts on which I offer this proposal, are furnished me by my colleague, Mr. Bill. His remedy is scarcely any other than strips of adhesive plaster, so applied over the crusts as to make an equable compression, except in very bad cases, where other means are used in addition to it. But as he has favoured me with his ideas on the subject, I shall take the liberty to subjoin them, as conveyed in his own words.

"If the case be a mild one, I direct the head to be cleared cleared of the hair, either with a pair of scissars, or by shaving it, and then well washed with chamomile tea, in which some soft soap has been dissolved. This done, I apply strips of Baynton's plaster over the whole head, so as to form a complete cap. The strips should be about fourteen inches long, and an inch and a half broad, and laid on in such a manner as to make an equal compression over the whole scalp. This application should be renewed every third or fourth day, each time previously washing and shaving the head. Whilst the patient is under cure, I commonly prescribe, if the subject be a child, five or six grains of the hydrargyri cum sulphure, either alone, or mixed with a little rhubarb.

"Provided the case be a bad one, of long standing, and such as has resisted the remedies usually employed, I have recourse to a more complicated mode of treating the disease, which, hitherto, has not disappointed my expectations. In the first place, I order as much of the hair to be cut off with scissars, as can be done without exciting severe pain; then I direct a large emollient poultice to be applied night and morning for a day or two; then the head to be shaved, and washed with the solution of soft soap in chamomile tea, and afterwards rubbed with an ointment composed of equal parts of the saturnine and mercurial nitrated ointments.

"These applications, the poultice excepted, I repeat every night and morning, for a few days; then I employ Baynton's strips, as before directed. In cases that are very inveterate, the ointment may be rubbed on the head previous to each application of the plasters, though I believe it is not always necessary. During the time these applications are making, I usually direct the patient to take of Plummer's pill, and of the decoction of the woods, with antimonial wine, and sweet spirit of nitre, enjoining at the same time a milk and vegetable diet.

"By persisting regularly in this plan of treatment, I have effected a cure in some bad cases in less than a month. However, I am by no means so sanguine as to expect that this nasty disorder will, in every instance, yield to these remedies; or that the disease so subdued will never relapse again; but I am confident that the method which I have pointed out, will, if properly pursued, generally succeed.

"In a great variety of cases I apprehend that nothing more will be necessary to obtain a cure, than a judicious application of the plasters, with a suitable diet, attention to cleanliness, and the state of the bowels. Yet, after after all, I would not have the plaster-bandage considered in any other light, than as a useful auxiliary in the cure of a troublesome complaint."

Mr. B. was led, as he informs me, by an easy transition, to the employment of the plaster bandage in tinea capitis, from his success with it in ulcers, and herpetic affections on the leg; and the justness of the analogy is confirmed by the most satisfactory result. As he has not himself indulged in any speculations in what manner the salutary effects are produced, I will here hazard a few thoughts on the subject.

If we consider the tinea capitis as a disease of local debility, (and in several of Mr. B's cases it was cured by the bandage alone, without the aid of internal remedies) the principle that governs the plaster bandage in the treatment of ulcers on the legs will, in the present instance, very closely apply. In this view I should be disposed to ascribe its active virtue to the mechanical principle of supplying tone, an early symptom of which is a good suppuration under the crusts; and this always indicates invigorated action, and a disposition to heal. Soon after the appearance of this favourable change, the crusts are cast off in quick succession, cicatrization is promoted, and finally this unmanageable disorder is both easily and speedily removed.

[To be continued.]

